# Authors’ Response to Peer Reviews of “Large Language Models for Pediatric Differential Diagnoses in Rural Health Care: Multicenter Retrospective Cohort Study Comparing GPT-3 With Pediatrician Performance”

**DOI:** 10.2196/73258

**Published:** 2025-03-19

**Authors:** Masab Mansoor, Andrew F Ibrahim, David Grindem, Asad Baig

**Affiliations:** 1 Edward Via College of Osteopathic Medicine, 4408 Bon Aire Dr, Monroe, LA, 71203, United States, 1 5045213500; 2 Texas Tech University Health Sciences Center School of Medicine, Lubbock, TX, United States; 3 Mayo Clinic, Rochester, MN, United States; 4 Department of Radiology, Columbia University Medical Center, New York, NY, United States

**Keywords:** natural language processing, NLP, machine learning, ML, artificial intelligence, language model, large language model, LLM, generative pretrained transformer, GPT, pediatrics

*This is the authors’ response to peer-review reports for “Large Language Models for Pediatric Differential Diagnoses in Rural Health Care: Multicenter Retrospective Cohort Study Comparing GPT-3 With Pediatrician Performance.”*

We thank the reviewers [1] for the thoughtful and constructive feedback on our manuscript, “Large Language Models for Pediatric Differential Diagnoses in Rural Health Care: Multicenter Retrospective Cohort Study Comparing GPT-3 With Pediatrician Performance” [2]. We are grateful for the opportunity to revise and improve our work based on the insightful comments provided. Below, we provide detailed responses to the reviewers’ comments and outline the changes made to the manuscript.

## Comments and Responses

- *Please clarify why GPT-3.5 or GPT-4 (instead of GPT-3) was not used despite being available at the time of the study.*

**Response:** Thank you for highlighting this point. We have clarified that GPT-3 (DaVinci version) was selected because it was the most advanced version available during the study period. The Discussion section now also highlights the potential benefits of GPT-3.5 and GPT-4 for future studies, particularly in addressing rare or complex diagnoses.

Action taken: Added a rationale for GPT-3 selection in the Methods (Model Training and Fine-Tuning) section and expanded on the potential of GPT-3.5 and GPT-4 in the Discussion (GPT-3 vs Newer Models) section.

- *Why were racial and ethnic demographics not included? (“Data distribution gaps: No comparison of racial identity distribution between training and testing sets. Please consider adding a table or section on these demographic comparisons to ensure representation across subgroups.”)*

**Response:** We acknowledge this limitation and have added a justification for the absence of this data. Specifically, the dataset lacked structured fields for racial or ethnic demographics due to its retrospective nature. We recommend future studies prioritize collecting this information to assess potential biases and ensure equitable performance.

Action taken: Added this explanation in the Materials and Methods (Participants and Data Collection) section.

- *Evaluation metrics: The study primarily uses specificity and sensitivity for evaluating large language model–generated responses, which may not capture the full quality of the outputs. Incorporating natural language processing metrics such as Recall-Oriented Understudy for Gisting Evaluation (ROUGE) and bilingual evaluation understudy (BLEU) can help assess the quality of generated responses more comprehensively. ROUGE measures the correspondence between the automatically generated response versus that of the human and what was expected. There are also issues associated with large language model generations of responses such as hallucination and the lack of attribution. Please specify or comment on how those and other issues were measured.*

**Response:** We have included a discussion on hallucinations—where models generate inaccurate or unsupported outputs—and their implications for clinical use. Suggestions for addressing these issues, including the use of natural language processing metrics (eg, ROUGE and BLEU) and physician feedback mechanisms, have been added to the Discussion (Practical Implications) and Future Directions sections.

Action taken: Added text addressing hallucinations and quality evaluation in the relevant sections.

- *Figure 1 is mentioned but not included in the article, which affects comprehension of the study design and findings. Please include Figure 1 or provide an alternative reference to explain the content of the missing figure. Figures are helpful for readers to quickly grasp complex methodologies and findings.*

**Response:** Thank you for this suggestion. We have created and included a flowchart (Figure 1) summarizing the study workflow, including data collection, preprocessing, training/testing split, model fine-tuning, and evaluation steps.

Action taken: Added Figure 1 to the manuscript and referenced it in the appropriate sections.

- *Lack of clarity on potential implementation in rural health care settings: The study could be strengthened by detailing how the artificial intelligence (AI) model might be implemented in rural health care settings, including the specific challenges involved. Key considerations include the need for sufficient infrastructure (eg, electricity, internet) and the necessity of training health care providers unfamiliar with AI tools. Additionally, discussing both the potential impact (eg, improved diagnostic efficiency) and limitations (eg, handling incomplete data or overreliance on AI) would provide a more comprehensive road map for deployment in rural environments.*

**Response:** We have elaborated on the challenges of implementing AI tools in rural health care, including infrastructure limitations (eg, internet access, power supply) and costs. Recommendations for subsidized programs and partnerships with technology providers have been added to address these barriers.

Action taken: Expanded the Discussion (Practical Implications) section.

- *Address the lower accuracy for rare diagnoses.*

**Response:** We agree with this observation and have emphasized the need for targeted fine-tuning using domain-specific datasets to improve performance on rare pediatric conditions. This point is now discussed in the Discussion (Rare Diagnoses) section.

Action taken: Added text on targeted fine-tuning for rare diagnoses.

- *Normality test: The study does not address whether data normality was assessed before statistical analysis. Determining the distribution of the data is key to selecting the appropriate statistical test to analyze such data. The Kolmogorov-Smirnov test could aid in understanding data distribution, specifically testing for normality. If the data is not found to meet normality criteria, nonparametric methods should be applied. Including a data normality assessment and explaining the choice of a particular statistical test would significantly strengthen the reliability of the study.*

**Response:** Added data normality assessment details to Statistical Analysis section, specifying Kolmogorov-Smirnov testing and justification for parametric methods.

- *Power analysis assumptions: The assumptions underlying the power analysis are unclear, particularly regarding how specific diagnoses affect this analysis. It is advised to elaborate on the power analysis methodology, including the rationale behind sample size choices and their implications for diagnosis variability.*

**Response:** Expanded power analysis methodology with sample size rationale and considerations for diagnosis variability.

- *Sample size and generalizability: The sample size of 500 encounters may not adequately represent the broader pediatric population, particularly in diverse settings. Furthermore, using data from a single health care organization limits the applicability of findings to other settings. These limitations should be discussed, particularly how the validity of the results might change when it is tested with data from other health care centers. If possible, authors should mention and cite studies that reported on this effect. Additionally, future studies should consider expanding the sample size through multicenter collaborations or including data from patients with more diverse demographics to validate results across different health care environments thereby enhancing generalizability.*

**Response:** Enhanced discussion of sample size limitations with specific references to performance decreases across datasets (5%-15%).

- *Cross-validation across organizations: The model’s reproducibility across various health care settings is not demonstrated. Evidence shows models often underperform with data from different sources. Including cross-organization validation and clearly acknowledging this limitation in the Discussion by citing relevant studies would enhance robustness. Furthermore, addressing this limitation in future work could pave the way for broader adoption and application of the model.*

**Response:** Added detailed Cross-Validation Limitations section citing studies showing model performance drops (12%-20%) across organizations.

- *Diagnostic exclusion or inclusion clarification: The preprocessing section does not clarify if physician diagnostics were included or excluded, leading to potential confusion for readers and impacting reproducibility. It would be helpful to know whether physician diagnostics were included in training and why. Clarifying this aspect would help standardize study replication and improve the study’s transparency.*

**Response:** Clarified that physician-generated diagnoses were from retrospective data, not prospectively collected.

- *Data and model specifics for replicability: The study would benefit from more thorough descriptions of dataset characteristics, fine-tuning model parameters, and preprocessing methods. For validation, consider adding multicenter dataset details. Adding this information would enable other researchers to replicate and build upon the study’s findings, thereby enhancing its scientific contribution.*

**Response:** Added comprehensive technical appendix with model specifications and implementation details.

- *Software and tools documentation: The authors describe using both Python (with scikit-learn) and IBM SPSS Statistics, but it is unclear what the software’s sources are. Specifying sources for Python and scikit-learn (eg, “Python 3.8 [Python Software Foundation, Delaware, USA]”) and clarifying the respective roles of Python and SPSS in the analyses would enhance transparency and allow for the reproducibility of the study.*

**Response:** Expanded Statistical Analysis section with rationale for test selection and metrics.

## Additional Revisions

- Included a detailed Table 1 legend to clarify evaluation metrics (eg, true positive, false positive, true negative, and false negative).
- Added a sentence in the Future Directions section emphasizing the need for training programs tailored to rural health care providers.
- Corrected minor typographical errors in tables and sections for clarity.
- Expanded Introduction with relevant literature on large language models in pediatric contexts, including recent studies by Ramesh, Ghosh, and Haddad.

We hope these revisions address the reviewers’ comments and improve the clarity, transparency, and quality of the manuscript. We sincerely thank the reviewers and the editorial team for their valuable feedback. Please do not hesitate to contact us with any additional comments or concerns.

## Abbreviations

AI: artificial intelligence
BLEU: bilingual evaluation understudy
ROUGE: Recall-Oriented Understudy for Gisting Evaluation
